# Global Sources and Pathways of Mercury in the Context of Human Health

**DOI:** 10.3390/ijerph14010105

**Published:** 2017-01-22

**Authors:** Kyrre Sundseth, Jozef M. Pacyna, Elisabeth G. Pacyna, Nicola Pirrone, Rebecca J. Thorne

**Affiliations:** 1 NILU-Norwegian Institute for Air Research, Department of Environmental Impacts and Economics, Instituttveien 18, P.O. Box 100, Kjeller NO-2027, Norway; jp@nilu.no (J.M.P.); ep@nilu.no (E.G.P.); rjt@nilu.no (R.J.T.); 2 AGH-University of Science and Technology, Department of Energy and Fuels, Krakow30-059, Poland; 3 CNR-Institute of Atmospheric Pollution Research, Area della Ricerca Roma 1, Via Salaria Km 29 300, Monterotondo (Rome) 00015, Italy; pirrone@iia.cnr.it

**Keywords:** mercury, global sources, pathways, emission scenarios, human health, policy-making

## Abstract

This paper reviews information from the existing literature and the EU GMOS (Global Mercury Observation System) project to assess the current scientific knowledge on global mercury releases into the atmosphere, on global atmospheric transport and deposition, and on the linkage between environmental contamination and potential impacts on human health. The review concludes that assessment of global sources and pathways of mercury in the context of human health is important for being able to monitor the effects from implementation of the Minamata Convention targets, although new research is needed on the improvement of emission inventory data, the chemical and physical behaviour of mercury in the atmosphere, the improvement of monitoring network data, predictions of future emissions and speciation, and on the subsequent effects on the environment, human health, as well as the economic costs and benefits of reducing these aspects.

## 1. Introduction

Mercury (Hg) is a persistent and toxic element that, through anthropogenic activities or natural processes, can be mobilized from natural deposits into the biosphere. It can travel long distances within air masses and water currents, undergo methylation into methylmercury (MeHg; CH_3_Hg^+^), and biomagnify and bio-accumulate in food chains to levels that can be dangerous to humans. Chronic exposure to methylmercury via consumption of fish and other marine species is a major concern for human health, especially developmental exposure that may cause neurological alterations [1,2,3,4,5,6,7]. Mercury exposure has also been proven to cause elevated risks for cardiovascular diseases and events, and in case of severe exposure, there is a risk of negative impacts to the reproductive and immune systems and premature death [8,9]. Recent studies conclude that rice consumption in some regions is a significant dietary source of prenatal methylmercury exposure, and that the scale of such exposure exceeds exposure from fish consumption [10]. At the same time, the inhalation of mercury vapour associated with artisanal and small-scale gold mining (ASGM) is a significant source of mercury exposure to humans in many developing regions [11]. It is, therefore, important to obtain knowledge on how mercury contamination on a global scale can be limited, and to identify suitable strategies for the prevention of adverse health outcomes related to mercury emissions. The international scientific community has contributed to this knowledge by investigating the behaviour of mercury in the environment [12,13] and its environmental [14,15,16,17,18,19,20,21,22,23,24], human health [9,25,26], and economic consequences [27,28]. Furthermore, the potential for climate change to affect the environmental transport of mercury and its consequent risks to human health, has gained increased attention during recent years [26,29,30,31]. The major conclusion drawn from the aforementioned studies is that international action is required as soon as possible to reduce mercury emissions and hence human exposure on local, regional, and global scales. The 2013 signing and subsequent implementation of the Minamata Convention is a response to this need, and it can be argued that scientific knowledge and analytical tools need to be in place to accurately assess global anthropogenic mercury emissions in the context of human health and for being able to verify the effects of actions encouraged by the treaty.

This paper reviews information from the existing literature and the EU GMOS (Global Mercury Observation System) project (http://gmos.eu/) to assess the current scientific knowledge on global mercury releases into the atmosphere, global atmospheric transport and deposition, and the linkage between environmental contamination and potential impacts to human health. The paper also discusses uncertainties and research needs associated with the modeling of current and future global sources and pathways of mercury in the context of human health.

## 2. Global Sources of Mercury Emissions

Mercury cycling among various reservoirs (atmosphere, lithosphere, biosphere, and hydrosphere) results both from anthropogenic and natural emission sources. Various global mercury assessments [17,32] have identified the atmosphere as the dominant pathway for the distribution of mercury on a global scale, although some of the atmospheric emitted mercury is locally deposited on terrestrial and aquatic surfaces. Mercury emitted in one part of the world may be transported to another part of the world, depending on the wind direction and speed, and the chemical behaviour of the mercury during atmospheric transport. Several transport and fate models investigate such transport and the exchange of mercury among global reservoirs, and the amount and source-attribution for mercury deposition [23,33]. The models rely on information of emission sources from direct anthropogenic emissions and from specific natural and re-emission processes.

Mercury existing naturally in the Earth’s crust is released via the weathering of rocks and from geological movements, such as from volcanoes and geothermal sources, and is normally referred to as a primary natural source. These sources tend to increase the global pool of mercury in the upper layer environment. Secondary natural sources redistribute mercury among and within ecosystems due to historically-emitted mercury (from natural or anthropogenic sources) on vegetation, land, or water surfaces. Such re-emissions result from land use changes, biomass burning, meteorological conditions, and exchange mechanisms of gaseous mercury at air–water, top soil, or snow–ice pack interfaces [13,17,20,32].

Mercury emitted from primary anthropogenic sources mainly derives from high temperature industrial processes, where it is introduced as a minor constituent in fuels, minerals, and wastes. It evaporates from raw materials during the high-temperature production of industrial goods and combustion of fuels, entering the ambient air with exhaust gases. The concentration of mercury as an impurity in raw materials, such as in fossil fuels and in various ores, physical and chemical properties of the mercury affecting its behaviour during the production process, the technology employed in industrial processes and the type and efficiency of emission control equipment, are regarded as the main parameters affecting the amount of mercury being emitted from anthropogenic sources [13,34]. Mercury emitted from secondary anthropogenic sources is generally emitted from intentional use in artisanal and small-scale gold production, in industrial process applications or in consumer products [17]. Anthropogenic sources are regarded as the main contributor to the global biogeochemical cycling of mercury by causing higher levels of re-emissions [11,35].

### 2.1. Anthropogenic Emissions

Global mercury emissions from anthropogenic sources have been inventoried in many studies over the years [17,35,36,37,38], which have become more advanced with time by involving a larger number of sources and more robust inventory methodology. Recent studies also include future emission scenario estimates [17,35,39,40], while some inventories have been methodologically harmonized for the purpose of trend analysis [41]. These global assessments and associated modelling have improved our understanding of mercury emissions from both primary and secondary sources, their transport and deposition, and have provided information on likely changes in the future due to varying levels of emission control under different scenarios. State of the art emission inventory results presented in the global mercury assessment prepared by The United Nations Environmental Programme (UNEP) and the Arctic Monitoring and Assessment Programme (AMAP), based on the methodology in Pacyna et al. [17], estimated anthropogenic mercury emissions globally in 2010 to about 1960 tonnes·year^−1^ with an uncertainty range of 1010–4070 tonnes·year^−1^. ASGM were identified as the major anthropogenic sources, accounting for 37% of the total anthropogenic mercury emissions to the global atmosphere, followed by combustion of coal and other fossil fuels for energy and heat production in power plants and in industrial and residential boilers, accounting for 25% of the total mercury emissions globally [13]. Other significant industrial sources of global mercury emissions to the atmosphere, were primary non-ferrous metals production (10%), cement production (9%), large-scale gold production (5%) and waste from consumer products (mostly landfill, but also incineration; 5%), contaminated sites (4%), pig iron production (2.3%), chlor-alkali industry (1.4%), oil refining (1.4), mercury production (0.6%), and dental amalgam emitted through cremation (0.2%) [13].

It was concluded already in the 1990s that Asian countries, particularly in Southeast Asia, such as China and India, dominate total global mercury emissions [17], although global anthropogenic mercury emissions to the atmosphere have appeared to be relatively stable between 1990 and 2010 [41]. Increased use of air pollution controls, removing mercury as a co-benefit in combination with some mercury-specific removing technologies has, during the time period, slowed down or, in some cases, decoupled emissions from the increased energy demand. This has been reported for Europe and North America, and seems now to be the trend for new coal-fired power plants with state-of-art pollution controls installed in China [13,17]. Nevertheless, a general increase in emissions in Asia is being observed, resulting from growing demands for energy in the region and increasing industrial production and the subsequent increase in fossil fuels for power and heat generation. Details on the impact of various factors on the magnitude of emissions from energy and industrial sources are presented in the literature [42].

### 2.2. Natural and Re-Emitted Emissions

The scientific literature has, over the years, presented several estimates on the annual magnitude of natural and re-emitted emissions, ranging from 3600 tonnes·year^−1^ [43] to 5300 tonnes·year^−1^ [44,45]. In the EU GMOS project, the inventory of primary natural emissions and re-emissions processes of historically-deposited mercury over land and sea surfaces were recently assessed to be 5207 tonnes·year^−1^ [26,33]. The estimate is comparable with the information provided in the latest work by Cohen et al. [23], estimated to 6500 tonnes·year^−1^, adopted from the literature [21,22,46,47,48]. The assessment during the EU GMOS project concluded that the oceans are the most important natural and re-emission sources, contributing 36% of the natural and re-emitted emissions of mercury, followed by biomass burning (9%), deserts, metalliferous and non-vegetated zones (7%), tundra and grassland (6%), forests (5%), and evasion after mercury depletion events (3%). Overall, the relative contribution of terrestrial surfaces is 2429 tonnes·year^−1^ and the contribution from surface waters 2778 tonnes·year^−1^, leading to a 5207 tonnes·year^−1^ total.

### 2.3. Future Emissions

Future mercury emissions are dependent upon many variables, including the development of national and regional economies, development and implementation of technologies for reducing emissions, and possible regulatory changes. In addition, the implementation of various climate change mitigation options to reduce carbon dioxide emissions (CO_2_), such as improvements of energy efficiency in power stations, replacement of fossil fuels by renewable sources, improvements of combustion and industrial technologies, and application of carbon capture and storage (CCS) technologies, are expected to cause co-benefit effects on mercury emission reductions [26,33].

**Figure 1:**
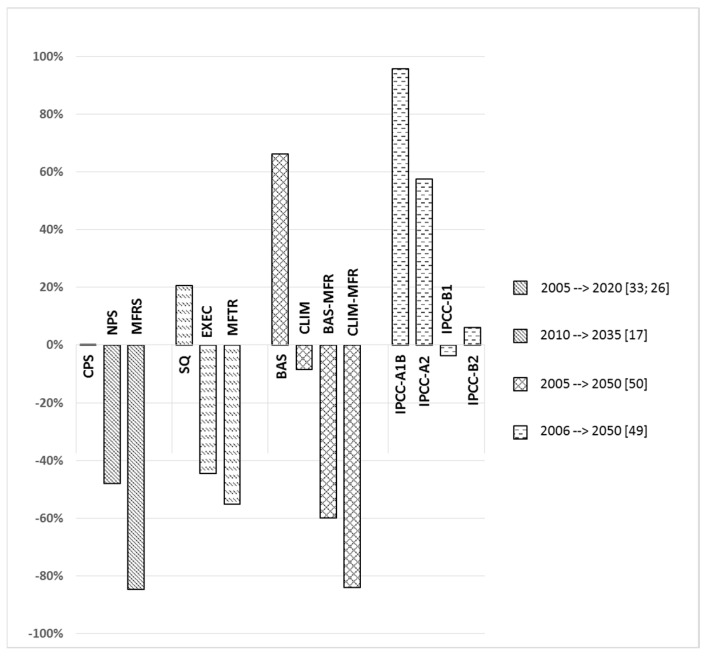
The estimated global change in anthropogenic mercury emissions under various scenario developments (relative to their base year).

Several global and regional projections on atmospheric mercury emissions have been presented in the literature [17,26,33,40,49,50,51] with various assumptions on temporal-, socioeconomic-, technology-, and policy developments. The estimated global change in anthropogenic mercury emissions under various scenario developments relative to their base year are presented in Figure 1.

Recent mercury emission estimates for the year 2035 projected for the Current Policy Scenario (CPS), New Policy Scenario (NPS), and Maximum Feasible Reduction Scenario (MFRS) developed in the EU GMOS project were presented in Pacyna et al. [33] and Sundseth et al. [26]. The scenarios were based on the recent AMAP/UNEP work [13] and were supported by international statistics, such as those from the International Energy Agency (IEA), including the World Energy Outlook (WEO) and Energy Technology Perspectives (ETP).

The CPS assumptions showed that if mercury continues to be emitted with the control measures and practices that are decided on in 2010 against a background of increasing population and economic growth, the emissions will remain approximately the same in 2035. An increased energy demand, especially in non-OECD countries will contribute to an increase in the total mercury emissions globally, mainly caused by the assumptions about future economic growth, but the implementation of additional control measures will result in a counter-effect. The NPS was estimated to result in a benefit of reducing global mercury emissions by almost 50% in 2035 (1020 tonnes·year^−1^ against 1960 tonnes·year^−1^) under the assumptions employed in the scenario, taking into account that policy commitments and plans announced by countries worldwide to reduce greenhouse gas emissions, in addition to phasing out fossil energy subsidies, would be fully implemented. The MFRS assumptions, i.e., that all counties reaching the highest feasible reduction efficiency in each emission sector, resulted in 85% less emissions (i.e., 1660 tonnes·year^−1^) than those envisaged under the CP scenario. Sector- and region-specific mercury emissions under the various emission scenarios can be observed in [35].

Compared to previous emission scenario estimates for the year 2020 as presented in Pacyna et al. [17], of taking action or not taking action to control emissions according to the status quo (SQ), the extended emissions control (EXEC), and maximum feasible technology reduction (MFTR) scenario, the more recent estimates for 2035 envision a more optimistic emission reduction course in both relative and absolute terms. This can, to some degree, be explained by observed worldwide policy developments and by more detailed information to support the assumptions on the implementation and use of traditional air pollution control devices (APCDs) and measures designed to prevent climate change and other environmental problems through energy efficiency and switching to lower carbon fuels or energy technologies [26,33].

In Rafaj et al., 2013 [50], scenarios for the year 2050 were developed according to the greenhouse gas and air pollution interactions and synergies (GAINS) model and defined under baseline with current national legislation (BAS), climate mitigation scenario with current legislation (CLIM), baseline with maximum feasible reduction (BAS-MFR), and climate mitigation scenario with maximum feasible reduction (CLIM-MFR) policies. The study found that large co-benefits in terms of reduced mercury emissions can be achieved from the parallel control of air quality and greenhouse gases (GHGs), and that maximum feasible reduction strategies can substantially reduce future mercury emissions from the base year to around the same endpoint of 2035. Compared to the MFRS scenario in [26,33], maximum feasible reductions were around the same level.

On a regional scale, Rafaj et al. [40] provided GAINS model projections for the year 2050 on the implications of renewable energy policies on atmospheric emissions of mercury in Europe. They found that current emissions of mercury in Europe are likely to remain at the same level under baseline conditions, but applying current standard air pollution control devices would mean a 35% reduction of mercury emissions. Low carbon policies and renewable electricity deployment also considered in this study led to a considerable co-benefit removal of mercury.

By investigating energy and fuel use patterns under the IPCC (Intergovernmental Panel on Climate Change) A1B, A2, B1, and B2 scenarios of development, Streets et al. [49] previously found it likely that global mercury emissions would increase until the year 2050 from the base year of 2006. The estimates mostly reflected a large increase in power sector and industrial emissions, especially in China.

Although the various scenarios results presented in the literature mainly differ in their assumptions on temporal-, socioeconomic-, technology-, and policy developments, the main difference in results seems to be caused by assumptions on the degree of abatement technology implementation, and their efficiency of mercury removal, as well as the extent of parallel control of air quality and greenhouse gas mitigation.

The effect future global scenarios have on relative emissions of the different mercury species has been investigated in some scenario assessments (i.e., [39,50]), although such analysis is very sensitive to combustion conditions and thus requires detailed point source information. It can be claimed that such information, along with information on relevant emission projections, is important for environmental modelling and for being able to assist decision-making and policy.

## 3. Atmospheric Transport and Deposition

### 3.1. Air Concentration Levels and Atmospheric Deposition of Mercury

Understanding the transport and deposition patterns of mercury in the atmosphere is critical for assessing the presence and future risk for human health and for identifying effective policy options at local, regional, and global scales. Monitoring and model simulations to assess global mercury transportation, deposition, and source-receptor relationships have been undertaken e.g., in the EU GMOS project. Pacyna et al. [33] recently presented the basis of the results obtained during the project, in the form of an assessment of atmospheric deposition of mercury worldwide simulated by the GLEMOS (Global EMEP Multi-media Modeling System) and ECHMERIT (ECHAM5, MERcury, ITaly) global chemical transport models. According to the assessment, global distributions of surface gaseous elemental mercury (GEM) concentrations showed a pronounced gradient of mercury concentrations between the southern and the northern hemisphere and elevated concentrations in major industrial regions, such as in East and South Asia, Europe, and North America. High mercury concentrations were simulated in some regions, such as North and South America, Sub-Saharan Africa, and Indonesia due to a high level of mercury emissions reported from ASGM. According to Pacyna et al. [33], these results show good agreement with measurement observations, since the discrepancy between the simulated and observed concentrations of GEM did not exceed a factor of 1.5.

A review of a new global, Eulerian version of the HYPSLIT-Hg (Hybrid Single-Particle Lagrangian Integrated Trajectory) model has recently been provided in Cohen et al. [23], simulating global transport and deposition of mercury to the Great Lakes with different model configurations. The study concluded that source-attribution estimates on mercury are strongly dependent on using correct information regarding emissions, speciation, atmospheric chemistry, and proximity to the emission source.

### 3.2. Future Change in Deposition Levels

Several global models have been applied for source apportionment of mercury deposition, taking into account future scenarios [19,21,44,47,52,53]. Projections of future changes in mercury deposition on a global scale were recently simulated by GLEMOS and ECHMERIT for the three emissions scenarios of 2035 discussed above, i.e., the CPS, NPS, and MFTS. The scenario simulations predicted a decrease of up to 30% of mercury deposition in Europe and North America, and up to a 50% increase in South and East Asia under the CPS. According to the NPS a decrease in mercury deposition similar to the CPS situation in Europe and North America was predicted over the whole of the globe, except for South Asia, where an increase in deposition of 10%–15% was expected due to the growth of regional anthropogenic emissions. Model predictions based on the MFRS demonstrated consistent mercury deposition reduction on a global scale of about 35%–50% in the Northern Hemisphere and of about 30%–35% in the Southern Hemisphere. Thus, the most significant changes in mercury deposition (both increase and decrease) during the next 20 years for all considered scenarios were expected in the northern hemisphere and, in particular, in the largest industrial regions, where the majority of regulated emission sources are located [31].

In Corbitt et al. [53] a global atmospheric model coupled to the surface reservoirs to quantify 2050 emissions projections based on four emissions scenarios developed by the IPCC, found that mercury deposition in 2050 will stay similar to the present-day levels for the best-case scenario but will increase for the other scenarios. The largest increase was also predicted in Asia (in China and, particularly, in India), mostly because of increased contribution from domestic emissions. Lei et al. [46] considered similar IPCC-based scenarios and found an increase in wet deposition by the year 2050 over the continental United States in all cases.

### 3.3. Climate Changes

Climate change effects on mercury have previously been studied [13,26,29,54], especially for assessing such effects on mercury contamination in the Arctic [26] due to the assumption that the Arctic is particularly sensitive to climate change, compared to other regions. Such studies have concluded that increased air temperature may lead to the acceleration in the loss of sea-ice cover, which leads to a warmer upper ocean and, consequently, to changes in the atmospheric connections between the Arctic and lower latitudes. Atmospheric mercury chemistry, including atmospheric mercury depletion events (AMDEs) may also be affected by changing temperatures and precipitation [30,55] and it is expected that increased temperatures would slow down the mercury deposition if the concentrations of Bromine (Br) are assumed to be the same. Using the Danish Eulerian Hemispheric Model (DEHM) system, Christensen et al. [56] modelled in a long temporal perspective the atmospheric deposition in the Arctic due to climate change for the year ranges 1900–1999, 2090–2099, and 2190–2199. They found that under climate change scenarios and no change in mercury emissions, 20%–40% higher mercury deposition rates were modelled over continental areas in the Arctic and near-Arctic compared to the present-day climate, and lower deposition rates directly to the Arctic Ocean, which were modelled as 20%–40% lower in 2090–2099 compared to 1990–1999 [56]. The changes in mercury deposition were expected to result from changes in the atmospheric chemistry of mercury that result from reduced sea-ice cover and increased concentrations of ozone in the troposphere that were forecasted under the climate change scenario.

## 4. Human Health Impacts

### 4.1. Human Exposure

The majority of human exposure and health risks associated with mercury are due to the consumption of marine and freshwater foods [57,58,59], although recent literature points out that rice consumption in some regions in Asia is a significant dietary source for prenatal methylmercury exposure (e.g., [10]). ASGM is poverty driven, and acts as a significant source of mercury exposure to humans in many developing regions, particularly in South America, Asia, and Africa [11]. A summary of current literature regarding the health effects of mercury among those working and/or living in or near ASGM communities is provided in [11].

The results of large scale epidemiological studies concerning child development and neurological disabilities in relation to in-utero exposure to methylated mercury in various fish eating communities around the world have been summarized in Castoldi et al. [5]. Based on the concentrations measured in human blood from Disko Bay (Greenland), Nuuk (Greenland), and Nunavik (Quebec, QU, Canada) during the years 1992–2007, populations exhibited mercury levels above the safety limit. The dietary surveys (from 1999 to 2002) in Northwestern Alaska were higher for fish consumers when compared to those of non-fish consumers [9]. The health research on mercury in the EU ArcRisk project (http://www.arcrisk.eu/) focused on compiling and examining available information on mercury exposure and health effects in population groups in the Arctic and in the Mediterranean region. A summary of the results of mercury levels measured in the EU ArcRisk cohorts [60] is presented in Sundseth et al. [26]. The EU ArcRisk study concluded that participants in the Greek cohort appeared to have the highest levels of mercury, while cohort members in Slovenia, Norway, and Finland had the lowest mercury levels. Children living in Ribera d’Erbe and Menorca (in Spain) were exposed in the same range as adults in Greece. It was also concluded that the consumption of fish, particularly locally caught, is a major source of mercury exposure to humans. However, mercury levels in fish can vary greatly according to species and origin, with wild fish containing higher average concentrations than aqua-cultured fish. Further, it was concluded that more studies are needed to evaluate the potential threat to European populations taking into consideration exposure to various mercury compounds and mixtures of stressors with similar end-points [26].

### 4.2. Future Environmental Impacts

As found in previous AMAP assessments of mercury fate and behaviour in the Arctic [6,13,30], there is no linearity in the relationships between the future changes of mercury emissions and changes of mercury concentrations bioaccumulated in fish and seafood. A similar conclusion was reached in research carried out in the Great Lakes region in North America [61] and on Snake Island, Lake Ontario [62]. While chemical recovery of the environment due to emissions reductions could be measured as short term process, the biological recovery of the environment will take a much longer time. Bioaccumulation processes for mercury in biota also depend on the structures present in the local food-webs, and it has been concluded that there are problems with our ability to estimate marine food-web bioaccumulation with state-of-the-art modelling tools [26]. Regional considerations are, thus, important for making meaningful predictions or evaluation of impacts of global mercury exposure. In Northern Canada, patterns of environmental change are different in the Eastern and Western Arctic [27]. Further, temporal trends of mercury bioaccumulation in monitored animal populations vary regionally within the circumpolar Arctic, with a clear west-to-east gradient in the occurrence of recent increasing mercury trends [63]. These trends may be driven by different mercury sources from long-range transport and/or climate change. At the same time, there are indications that the signal in fish does not relate to the emissions, but rather to climate variables.

## 5. Final Discussion—Uncertainties and Research Needs

### 5.1. Global Sources of Mercury Emissions

**Figure 2:**
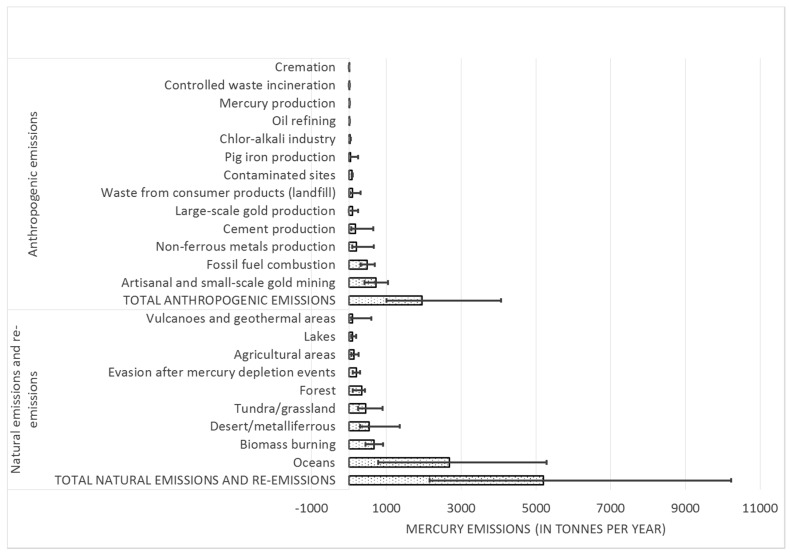
Global sources of mercury emissions with uncertainties. Based on [13,17,33].

By considering their upper and lower bounds as reported in several published papers and international reports (e.g., [36,64]), the range of uncertainty can be established for global natural and re-emitted mercury. To assess the uncertainty from global anthropogenic sources, the crude approach that has been presented in the AMAP/UNEP work [13] can be followed. An overview of the current global mercury emission sources and their uncertainties can be observed in Figure 2.

The uncertainty in the estimates is large for both anthropogenic, natural, and re-emitted emission sources, and it can be claimed that these amounts can be difficult to accept for decision-making purposes. The major components of uncertainties associated with anthropogenic emission sources have been assessed in global emission inventories [17], and found to result from a variety of factors, such as the collection of nationally-reported emissions data, the compilation of activity data, estimated emission factor data, and information about technology profiles and their mercury abatement efficiencies. In general, it has been identified that uncertainties associated with emission factors are considerably larger than those associated with the compilation of activity data. A summary discussion of the various uncertainty factors is provided in [13].

According to the literature [13,17], the most recent global emission inventories are complete and accurate for some anthropogenic sources. The energy and industrial sources exhibit, in general, lower uncertainties than for the emission inventories for waste incineration and ASGM. The study by Pacyna et al. [17] exhibits uncertainties of about 25% for energy and industrial sources, but up to a factor of three for waste incineration and ASGM. Higher emission ranges are estimated in the AMAP/UNEP work [13].

As a result of uncertainties associated with emission factors, there is a need for the development of improved information on the application of technology in industrial processes and the type of technology applied that reduce mercury emissions, as well as information on how these applications differ for various point sources. Improved information is needed regarding fuel use, including characteristics of coal use for power generation, in residential and commercial units, and on the mercury content of ores and concentrates used as inputs in industrial production. The chemistry of emissions from different sources also needs to be considered, since these will influence the scale and range of mercury transport on local, regional, and/or global levels. Nevertheless, it has been pointed out [33] that major improvements are expected in global emission inventories for anthropogenic sources, resulting from the push for countries to fulfil the requirements for the Minamata Convention.

Further, the high levels of emissions estimated from the ASGM sector that are not verified with field measurement data from areas of artisanal production of gold activities is an uncertainty issue. There is clearly a need for increasing the level of confidence in the assumptions regarding mercury emissions and releases from this sector.

It is also important to acknowledge that existing emission estimates for natural sources and re-emissions vary by a factor of three. This indicates that more measurements are needed, in particular for improving the accuracy of re-emitted mercury [33].

### 5.2. Atmospheric Transport and Deposition

The literature [24] points out that atmospheric mercury transport and deposition models based on global emission inventories have high associated uncertainties, and poor understanding of emissions from natural emissions and ASGM, is particularly an issue. Furthermore, emissions from biomass burning and legacy impacts of anthropogenic emissions are not adequately considered in the global mercury cycle. The re-emission processes in global models are usually assumed to be in the form of a static, one-way upward flux that simplifies the global biogeochemical cycle of mercury, and is, as such, a limitation of analysis [33].

Other issues with current models are their insufficient consideration of the speciation of mercury in the atmosphere, and use of limited data for atmospheric gaseous oxidized mercury (GOM) and dry deposition in their verification. However, recent comparison studies [31] have showed that current models are able to simulate distributions of gaseous reactive mercury (GEM) air concentration and total mercury deposition on a global scale, and are verifiable by comparing model estimates with ground-site measurements.

Gustin et al. [24] points to the need and importance of developing a measurement infrastructure to enable traceable and comparable assessments of atmospheric mercury and deposition, and to support these through measurements of mercury in biota.

### 5.3. Climate Change

Even though the actions following future policies and climate change mitigation are expected to reduce emissions and most likely the long-range transport of mercury, it is not clear how important these emission reductions will be compared to factors impacted by climate change. As summarized in Sundseth et al. [26], previous research has indicated that climate change can alter future levels of mercury concentrations and deposition through changes to vegetation cover and atmospheric oxidants, increased wildfires, enhanced air–seawater exchange, etc. [27,29]. In addition, climate change may affect several physical factors, such as those related to long-range transport from wind direction, precipitation rates, ocean currents, melting of polar ice caps and mountain glaciers, higher frequency of extreme events, and biotic transport.

Based on observed effects from recent climate warming on the Arctic’s physical environment, Stern et al. [29] concluded that the most important impacts regarding mercury occurs in precipitation rates and the type of precipitation (i.e., rain or snow), river discharge and seasonality, lake ice and sea ice seasonality, thickness and extent, declining length and depth of snow cover, increasing active layer depth in permafrost soils, altered vegetation and drainage basins, and changing atmospheric connectivity between the Arctic and southern latitudes [29].

Mercury methylation concentrations in the environment may increase with higher temperatures from higher bacterial activity in sediments [30]. On the contrary, microbial processes can lead to demethylation from higher temperatures but, in general, it is expected that the net methylation rate increases with higher temperatures. Since global warming is likely to extend the ice-free season, the season for mercury methylation is also increased at some geographic locations.

As concluded in [26], no current scientific information clearly answers whether climate change will increase or decrease the risk of exposure to mercury. Thus, more research is needed to further analyse the issue of climate change impact on the effects of mercury on human health.

### 5.4. Human Health Impacts

When it comes to mercury in the context of human health, it is important to acknowledge that methylmercury exposure not only depends on contamination levels, but also on dietary habits and food availability. The global risk of exposure will, therefore, to a large degree, depend on nutritional transition and food supply. It can be claimed that the amount of methylmercury ingested is becoming more uniform worldwide since there will be more global trade in fish and seafood. As discussed in [26] it is difficult to assess how mercury intake through fish and seafood consumption will change in the future, especially under climate changing conditions.

As pointed out in Driscoll et al. [20], the evaluation and management of human health risks will require a quantitative understanding of pathways and mechanisms that affect the transport of mercury from sources to ecosystems, the conversion of elemental mercury to methylmercury, and its bioaccumulation in food webs.

## 6. Conclusions

- Assessing global sources and pathways of mercury in the context of human health is considered important for being able to monitor the effects from the implementation of the Minamata Convention targets.
- Anthropogenic mercury emissions globally have been estimated to about 1960 tonnes·year^−1^ in 2010. Artisanal and small-scale gold mining (37%) and combustion of coal and other fossil fuels for energy and heat production in power plants and in industrial and residential boilers (25%) were identified as the major global anthropogenic emission sources to the atmosphere. Primary natural emissions and re-emissions processes of historically-deposited mercury over land and sea surfaces has recently been assessed to the amount of 5207 tonnes·year^−1^. The oceans are the most important natural and re-emission sources, contributing 36% of the natural and re-emitted emissions of mercury.
- Future mercury emissions are dependent upon many variables, including the development of national and regional economies, development and implementation of technologies for reducing emissions and possible regulatory changes. These can, to a large degree, be reduced as a co-benefit from the use of air pollution control devices, from reducing greenhouse gas emissions, and through the use of targeted mercury emission controls.
- Atmospheric mercury transport and deposition models based on global emission inventories have high associated uncertainties from poor understanding of emissions from natural emissions, artisanal and small-scale gold mining, biomass burning, and legacy impacts of anthropogenic emissions.
- No scientific information currently available is in the position to clearly answer whether climate change will increase or decrease the risk of global human exposure to mercury.
- New research is needed on the improvement of emission inventory data, the chemical and physical behaviour of mercury in the atmosphere, the improvement of monitoring network data, predictions of future emissions and speciation, and on the subsequent effects on the environment, human health, as well as the economic costs and benefits of reducing these aspects.
